# Orthodontic approach to treat complex hypodontia using miniscrews in a growing patient

**DOI:** 10.1590/2176-9451.20.4.082-090.oar

**Published:** 2015

**Authors:** Renato Barcellos Rédua, Paulo Cesar Barbosa Rédua, Carlos Eduardo de Almeida Ferreira, Adriana de Oliveira Lira Ortega

**Affiliations:** 1 Professor of Pediatric Dentistry, Escola Superior São Francisco de Assis (ESFA), School of Dentistry, Santa Teresa, Espírito Santo, Brazil; 2 MSc in Physiological Sciences, Universidade Federal do Espírito Santo (UFES), Vitória, Espírito Santo, Brazil; 3 Assistant professor, State University of New York, Buffalo, USA; 4 Postdoc in Oral Pathology, Universidade de São Paulo (USP), School of Dentistry, São Paulo, São Paulo, Brazil

**Keywords:** Anodontia, Orthodontic anchorage, Orthodontic space closure

## Abstract

This article reports orthodontic treatment of a case of hypodontia of five premolars in an 11-year-old female patient with a positive tooth size-arch length discrepancy in both dental arches. The patient had a straight profile with balanced facial growth. Setup manufacture revealed the possibility of achieving ideal occlusion by mesializing permanent molars up to 15 mm, in addition to keeping a primary molar in the dental arch. With the aid of absolute anchorage, the proposed mechanics was performed and the occlusion predicted in the setup was achieved, while profile and facial growth pattern were maintained. The use of miniscrews for extensive orthodontic movements was successful. Furthermore, one primary molar was extensively mesialized. The indication of gingivoplasty to correct gingival smile proved effective. This is considered a useful technique for orthodontists.

## Abstract (pt)

Este artigo apresenta o tratamento ortodôntico de um caso com hipodontia de cinco pré-molares, em uma paciente, de 11 anos de idade, com discrepância positiva de modelo em ambas as arcadas. A paciente apresentava perfil reto, com crescimento facial equilibrado. Por meio da confecção de *set-up*, verificou-se a possibilidade de se estabelecer uma oclusão ideal por meio da mesialização, de até 15mm, dos molares permanentes e manutenção de um molar decíduo no arco. Com o auxílio de ancoragem absoluta, foi realizada a mecânica proposta, alcançando-se a oclusão prevista em *set-up*, além da manutenção do perfil e do padrão de crescimento facial. A utilização de mini-implantes para grandes movimentos ortodônticos foi favorável, incluindo a extensa mesialização de um molar decíduo. A indicação da gengivoplastia para correção do sorriso gengival se mostrou acertada, sendo essa uma técnica de grande auxílio à Ortodontia.

## INTRODUCTION

Tooth agenesis is the most common developmental anomaly in humans, often representing a major clinical problem.^1^ Congenitally missing teeth are classified according to the number of missing teeth, except for third molars. Hypodontia is the term used to describe patients with agenesis of one to five teeth; however, when six or more teeth are missing, the condition is classified as oligodontia, whereas anodontia means all teeth are missing.^2,3^

The incidence of hypodontia of permanent teeth varies widely, and can be found in 2.6% to 11.3% in the overall population, while the incidence in primary teeth is considerably lower.^1^ Once congenital absence of one primary tooth occurs, that tooth successor is bound to be missing, given that the germ of the permanent tooth is formed from the germ of the primary tooth. Oligodontia is a rather rare condition, affecting about 0.1% to 0.2% of the population. It can occur as the manifestation of a syndrome or as an isolated condition linked to mutations in the MSX1 and PAX9 genes.^2,3^

Hypodontia features a wide range of manifestations. Depending on the number and location of missing teeth, it can affect esthetics, masticatory function, speech and occlusion balance, either through unwanted occlusal contacts, extrusion of antagonists, or inclination of teeth adjacent to the sites of missing teeth.^4^ Changes in size and shape are also commonly observed in teeth of patients with hypodontia.^5^

Complexity of hypodontia treatment varies widely, and it is more critical among young, growing patients whose psychological aspects and facial development are often compromised, thereby requiring a multidisciplinary approach.^5^

The aim of this article is to report the treatment of a growing patient presenting gingival smile and five premolars missing, and whose chief complaint was her unfavorable esthetics due to the size and diastemata of her anterior teeth.

## CASE REPORT

**Figure 1.**
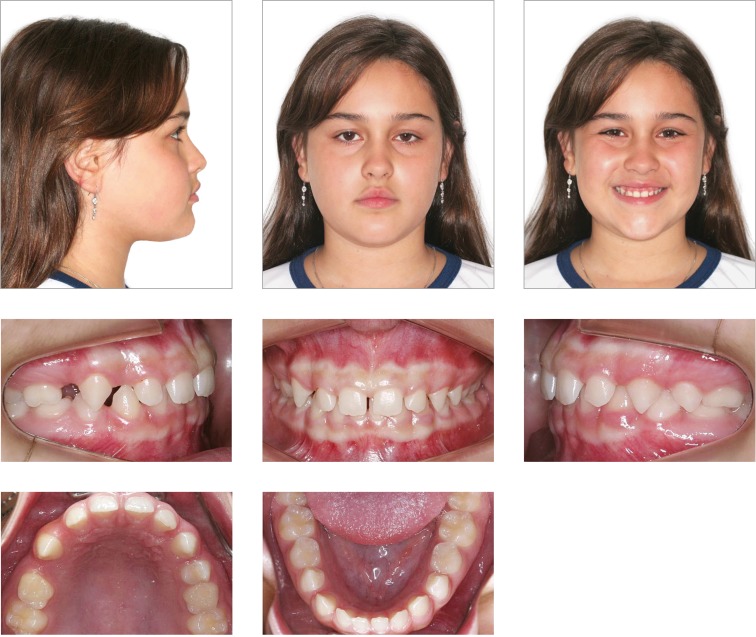
Initial orthodontic records.

This clinical case involves a female Caucasian 11-year-old patient who presented for treatment at private practice. She was referred to orthodontic treatment by a pediatric dentist who noted hypodontia involving five missing teeth. The patient reported as chief complaint generalized diastemata; and as secondary complaint, the small size of teeth (Fig 1).

**Figure 2.**
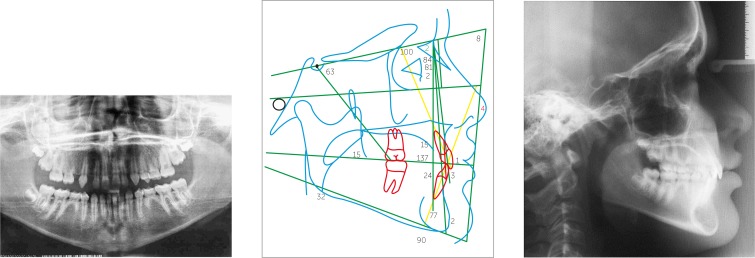
Panoramic radiograph confirms hypodontia of teeth #14, 15, 25, 35 and 45. Lateral cephalogram and cephalometric tracing reveal good axial inclination of maxillary and mandibular incisors, and balanced facial growth pattern.

The patient was at the beginning of pubertal growth spurt and in mixed dentition. She still had primary second molars and tooth #14 was missing. Radiographic examination confirmed that teeth #14, 15, 25, 35 and 45 were also missing, and in primary second molars, root resorption was not noticeable (Fig 2). No family history of hypodontia was reported, nor any signs or symptoms suggesting temporomandibular disorders.

Facial analysis revealed a relatively favorable growth pattern with balanced lower face dimension, normal nasolabial angle and lip competence, although both upper and lower lips were retrusive. At smiling, the patient presented with 6 mm of gingival smile, and reduced cervicoincisal dimension of maxillary canine and incisor crowns (Fig 1 and Table 1).

**Table 1.** Initial and final cephalometric measures.

	Initial	Final
SNA	84^o^	81^o^
SNB	81.3^o^	79^o^
ANB	2.7^o^	2^o^
1/./1	137^o^	165^o^
1/.NA	15^o^	5^o^
1/-NA	1 mm	1.3 mm
/1.NB	24^o^	6.9^o^
/1-NB	3 mm	1.3 mm
IMPA	87^o^	71^o^
SN.Go-Gn	32^o^	35^o^
Line H	4 mm	9 mm

Examination of lateral cephalogram and cephalometric tracing showed that upper and lower incisors were upright, there was balanced anteroposterior growth of the maxilla and mandible, with 2 degrees of ANB, as well as balanced vertical growth with 32 degrees of mandibular plane (Fig 2).

The patient also presented with deep overbite, coincident midlines and permanent molars in Class I relationship (Fig 1).

## Treatment goals

Treatment goal was to enhance esthetics and achieve appropriate occlusal function by eliminating generalized diastemata in both arches; extracting primary second molars, except for tooth #55; mesializing permanent molars and tooth #55; establishing normal overbite and Angle Class I relationship with tooth #55 taking the position of tooth #14. Furthermore, the therapeutic goals were to reduce gingival smile and increase the cervico-occlusal dimension of maxillary incisors and canines while keeping the axial inclination of incisors and preserving facial growth pattern and facial profile.

## Alternative treatment

To close the diastemata of incisors and canines, one alternative would be to increase the size of teeth by means of direct or indirect restorations, considering extensive oral rehabilitation without the aid of orthodontic treatment. One way to correct the congenital absence of teeth #14, 15, 25, 35 and 45 would be to keep primary second molars in the dental arch, maintain the space left by tooth #14, wait for the patient to stop growing, and then place an implant in the region of tooth #14. Finally, one should monitor primary second molars in case four more implants were needed in the region of second maxillary and mandibular premolars.

Closing the spaces left by missing teeth without the aid of absolute anchorage was not an option, given that incisor retraction was contraindicated, since incisors exhibited good axial inclination.

## Treatment plan

Patient's legal guardians opted for treatment with extraction of teeth #65, 75 and 85, and space closure by molars mesialization. Treatment plan included preadjusted orthodontic appliance placement followed by alignment and leveling, closure of diastemata between incisors and canines, four miniscrews placement, molar mesialization and referral for gingivoplasty.

## Treatment progress

Initially, composite resin stops were fabricated and placed on the occlusal surface of teeth #36 and 46 to correct overbite and obtain disocclusion, thereby allowing a fixed orthodontic appliance to be bonded to the lower arch.

Roth prescription 0.022 x 0.028-in brackets were bonded to incisors, whereas MBT prescription brackets were bonded to canines, premolars and molars. Molars were banded, with the exception of tooth #55 which received a standard attachment; and teeth #65, 75 and 85 which did not receive any attachments. After bonding the appliance, alignment and leveling were performed with 0.014-in nickel-titanium archwires, followed by 0.016-in, then 0.018-in and 0.020-in stainless steel archwires. When 0.020-in stainless steel archwires were used, the diastemata between incisors and canines were closed with the aid of elastomeric chains, so as to allow placement of miniscrews on the distal surface of canines.

The periodontist was shown the sites of choice for placement of four miniscrews (Fig 4) which were installed concurrently with the extraction of teeth #65, 75 and 85.

**Figure 3.**
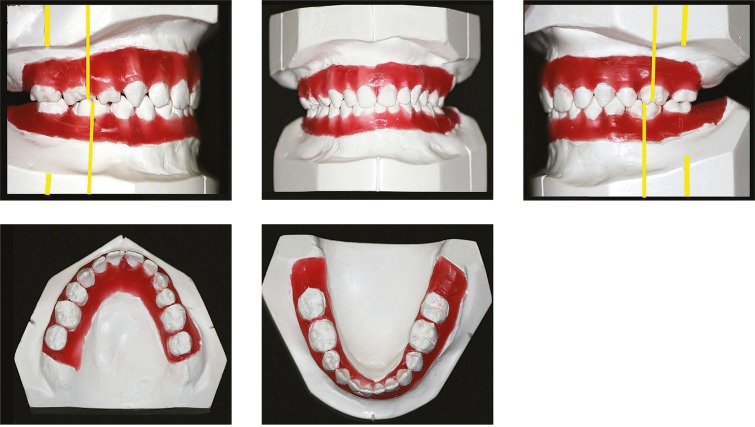
Setup used to measure the amount of mesialization required and to assess occlusal balance required for orthodontic treatment finishing.

**Figure 4.**
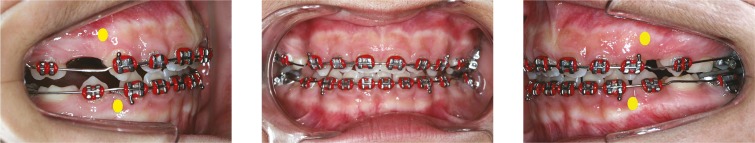
Photograph depicting four miniscrew placement sites chosen after closure of diastemata between incisors and canines was achieved.

**Figure 5.**
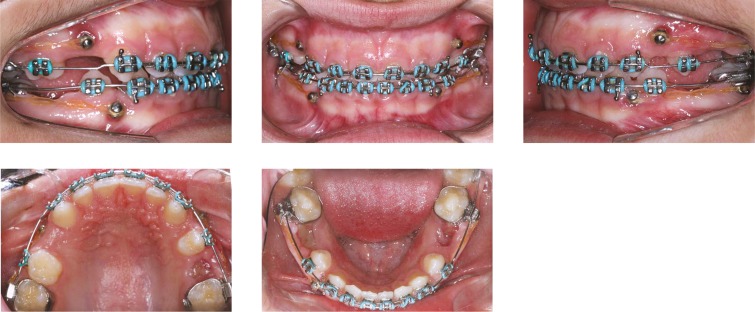
Placement of four miniscrews and mechanics carried out by means of elastomeric chains supported by a 0.020-in stainless steel archwire.

**Figure 6.**
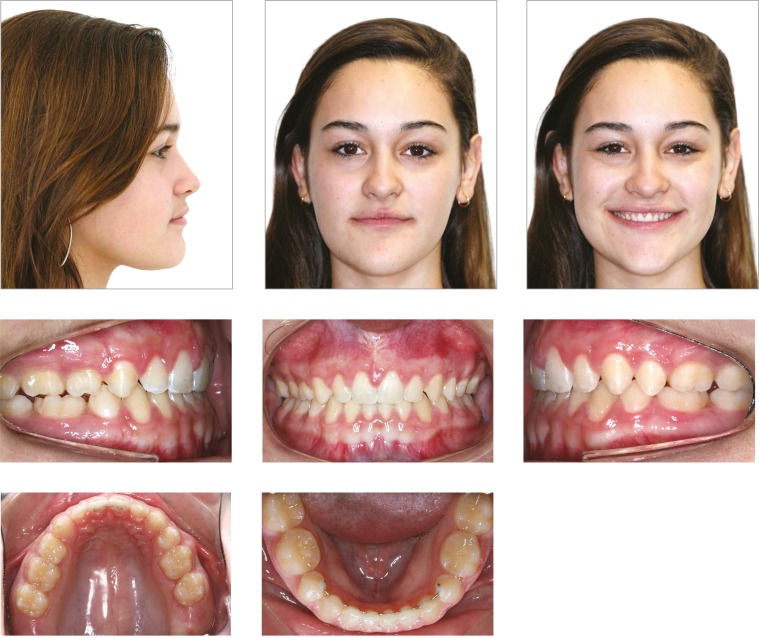
Post-treatment orthodontic records and gingivoplasty of teeth #13 to 24.

A 15-mm mesialization of molars was carried out using elastomeric chains supported on the miniscrews and by means of applying 200 g of force on each side, with molars sliding along a 0.020-in stainless steel archwire (Fig 5). The elastics were replaced every four weeks, on average. After closing the remaining spaces, a pair of 0.019 x 0.025-in rectangular archwires was placed to establish the correct torques, and dental intercuspation was achieved using intermaxillary elastics with a vertical component. It took seven months to align and level both dental arches and close the diastemata between incisors and canines, whereas molar mesialization spanned 25 months. Moreover, it took four months to finish treatment and establish balanced occlusal contacts, thereby totaling three years of orthodontic treatment. Gingivoplasty was performed after total closure of spaces, and before the case was finished (Fig 6).

A 0.018-in retainer was bonded to the lingual surface of all teeth from #34 to 44. On the upper dental arch, a retainer of the same gauge was bonded from tooth #11 to 21. Furthermore, a wraparound removable retainer was installed.

After treatment completion, an excellent cosmetic effect was achieved thanks to correction of gingival smile, increased cervico-occlusal dimension of maxillary incisors and canines, and closure of diastemata. A good functional result was achieved with deep overbite correction, closure of remaining spaces in the dental arch, molars in Angle Class I relationship, canines in ideal type I occlusion and coincident midlines. However, incisor axial inclination was not achieved (Fig 6).

## DISCUSSION

Diagnosis of dental anomalies is usually performed by pediatric dentists, as these professionals are the first to interact with children and adolescents.^6^ In the case presented herein, the patient was enrolled in a half-year prevention program in the private practice of a pediatric dentist. Tooth agenesis was detected when she was seven years old, after preventive analysis of panoramic radiograph, and her parents were informed accordingly. The patient was referred to orthodontic assessment and decided to wait until the final phase of mixed dentition before allowing orthodontic intervention.

During diagnosis and planning, patient's chief complaints were addressed. In assessing patient's vertical growth pattern, it was found that her gingival smile did not stem from excessive vertical growth of the maxilla. Therefore, she was referred to periodontal plastic surgery. Studies on smile esthetics show that a gingival smile not greater than 3 mm is perceived as esthetic by laypeople and dentists alike, whereas a 4-mm gingival smile is not considered esthetic by these groups, which underscores the need to address this condition in the case presented herein.^7,8^

One of the therapeutic options to close anterior diastemata would be to augment mesiodistal crowns with composite resin on anterior teeth by means of oral rehabilitation without resorting to orthodontic treatment, which had been proposed by another professional. However, patient's legal guardians only allowed closure to be effected orthodontically. The decision to close spaces by means of orthodontic therapy and not by increasing the size of teeth is more favorable biologically, since it eliminates the need for periodic replacement of restorations, avoids the risk of fractures in restorations, while also reducing financial costs.

In evaluating the alternatives to address congenital missing teeth, the authors chose to close spaces by means of molar mesialization instead of installing four dental implants. Such choice was based on the possibility of establishing a balanced occlusion without the need for implants, which entails a higher relative biological cost; increased financial costs, given the surgical costs and fabrication of crowns; the need for periodic replacement of crowns, and mainly because the patient was still growing, which might hinder a prompt resolution of the case. Auto-transplantation of third molars to the sites of second premolars was not an option, given that the stage of development of third molars was not sufficient. Additionally, a mandibular third molar was missing, and there was the risk of third molar resorption after auto-transplantation.^9^

A diagnostic setup was developed to measure the amount of mesialization required, in addition to checking the feasibility of attaining occlusal balance by keeping the maxillary primary second molar in the arch, as both premolars in the right upper quadrant were missing. Patient's parents were informed about the risk of root resorption of this primary molar due to the extensive orthodontic movement planned for the case. This could have eventually resulted in the loss of this tooth and the need to replace it with an implant after the patient stopped growing. Orthodontic diagnostic setups are extremely useful tools which aid in planning and performing orthodontic treatment. In this particular case, the end result was quite similar to the result predicted on the setup.^10^

As regards the unusual occlusal relationship of maxillary primary second molar which occluded with mandibular first permanent molar and mandibular first premolar, the setup showed that this relationship would allow a balanced occlusion. This was confirmed after treatment was completed, when a favorable occlusion with balanced distribution of forces in the vertical, lateral and anteroposterior directions became apparent.^11^

Roth prescription was used on incisors with a view to facilitating the incorporation of palatal and lingual torque in incisors, while the use of MBT prescription on canines and premolars is justified by the constant need for offsets in mandibular canines when Roth prescription is used.^12,13^ The use of MBT brackets on incisors would also be indicated for this clinical case.^12^^,^ 13 It should be noted that the use of preadjusted appliances is a personal choice and does not directly influence the success of the mechanics applied, given the constant need for individualization of cases during treatment finishing, as in the case described herein. Palatal and lingual torque were applied to upper and lower 0.019 x 0.025-in stainless steel archwires in order to reduce excessive incisor uprighting, but the results failed to meet expectations. The use of rectangular archwires, when closing the diastemata between canines and incisors prior to placing the miniscrews, could have reduced retroclination of maxillary and mandibular incisors.

Taking into account patient's good facial profile and good buccopalatal and buccolingual inclination of incisors, closing spaces by means of incisor retraction was ruled out, as it could have resulted in loss of lip support, thereby hindering facial esthetics. Thus, the use of absolute anchorage for molar mesialization was indicated. This absolute anchorage could be provided either by miniscrews or Bollard miniplates. The choice for miniscrews prevailed, given that they are easier to install and more affordable.^14,15^

According to Marassi and Marassi,^16^ mass retraction requires a force of 150-300 cN on each side (1 Newton = 100 cN = 102 g) which is equivalent to 150 to 300 g. This level of force is sufficient to close 0.5 to 1 mm space per month, considering normal alveolar bone height.^15,16^ Therefore, a 36-month treatment period was regarded as quite satisfactory, considering that it took 25 months for permanent molars to mesialize 15 mm.

When miniscrews are used as anchorage during sliding mechanics, super-elastic nickel-titanium springs, conventional nickel-titanium springs, elastomeric modules and elastomeric chains can be used.^16^ Although nickel-titanium springs are recommended due to their lower force variation, the patient complained of discomfort in the gingiva when the springs were being adapted; thus, the use of elastomeric chains was preferred.

Molar mesialization with the aid of elastic chains was preferred, as these afford continuous force for at least 21 days.^17^ Molar sliding was achieved by round 0.020-in archwires, since there was no need for torque control of incisors. Moreover, this archwire produces less friction than a 0.019 x 0.025-in rectangular archwire.

**Figure 7.**
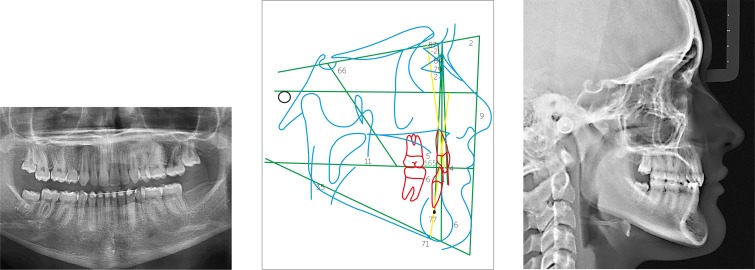
Panoramic radiograph reveals satisfactory root parallelism and total space closure. Lateral cephalogram and cephalometric tracing reveal good axial inclination of maxillary and mandibular incisors, maintenance of facial growth pattern and significant development of the chin.

After treatment, panoramic radiograph revealed good root parallelism of all teeth (Fig 7). Excessive uprighting of maxillary and mandibular incisors was observed in the final cephalometric tracing (Fig 7). This unfavorable situation may have been due to closure of anterior diastemata when determining miniscrew sites. The degree of esthetic commitment caused by the axial inclination of incisors on the face is debatable, especially considering that patient's chin was significantly augmented (Fig 7 and Table 1).

A 0.018-in retainer was bonded to the lingual surface of all teeth from #34 to 44 due to a greater potential for open spaces to relapse, given the vast amount of spaces remaining in the lower arch. A 0.018-in retainer was bonded to teeth from #11 to 21, and a removable wraparound plate was installed to maintain the transverse dimension as well as to close spaces. It is recommended that the lower retainer remain bonded indefinitely, both to avoid opening spaces and to prevent a decrease in intercanine width, which results from aging of the occlusion and may cause esthetic impairment. The patient was instructed to wear the plate for a period of 24 months, during which time the periodontal ligament could replace its fibers.^18^^,^ 19

Twelve months after removal of appliances, occlusal adjustment was performed by removing occlusal interference and verifying the absence of fremitus in incisors, thereby allowing removal of the upper retainer.

## CONCLUSIONS

Treatment of hypodontia often requires that osseointegrated dental implants be used to replace missing teeth. In this clinical case, a conservative proposal was presented for correction of five missing teeth without the need for implants, with satisfactory esthetic and functional results.

The use of miniscrews for extensive orthodontic movement was effective. Furthermore, a primary molar was satisfactorily mesialized. The indication of gingivoplasty to correct gingival smile proved effective, and considered a useful technique for orthodontists.
